# The impact of routine data quality assessments on electronic medical record data quality in Kenya

**DOI:** 10.1371/journal.pone.0195362

**Published:** 2018-04-18

**Authors:** Veronica Muthee, Aaron F. Bochner, Allison Osterman, Nzisa Liku, Willis Akhwale, James Kwach, Mehta Prachi, Joyce Wamicwe, Jacob Odhiambo, Fredrick Onyango, Nancy Puttkammer

**Affiliations:** 1 International Training and Education Center for Health (I-TECH), Nairobi, Kenya; 2 International Training and Education Center for Health (I-TECH), Seattle, WA, United States of America; 3 Department of Epidemiology, University of Washington, Seattle, WA, United States of America; 4 Department of Global Health, University of Washington, Seattle, WA, United States of America; 5 U.S. Centers for Disease Control and Prevention, Nairobi, Kenya; 6 National AIDS and STI Control Programme, Ministry of Health, Nairobi, Kenya; 7 Elizabeth Glaser Pediatric AIDS Foundation (EGPAF), Nairobi, Kenya; The Ohio State University, UNITED STATES

## Abstract

**Background:**

Routine Data Quality Assessments (RDQAs) were developed to measure and improve facility-level electronic medical record (EMR) data quality. We assessed if RDQAs were associated with improvements in data quality in KenyaEMR, an HIV care and treatment EMR used at 341 facilities in Kenya.

**Methods:**

RDQAs assess data quality by comparing information recorded in paper records to KenyaEMR. RDQAs are conducted during a one-day site visit, where approximately 100 records are randomly selected and 24 data elements are reviewed to assess data completeness and concordance. Results are immediately provided to facility staff and action plans are developed for data quality improvement. For facilities that had received more than one RDQA (baseline and follow-up), we used generalized estimating equation models to determine if data completeness or concordance improved from the baseline to the follow-up RDQAs.

**Results:**

27 facilities received two RDQAs and were included in the analysis, with 2369 and 2355 records reviewed from baseline and follow-up RDQAs, respectively. The frequency of missing data in KenyaEMR declined from the baseline (31% missing) to the follow-up (13% missing) RDQAs. After adjusting for facility characteristics, records from follow-up RDQAs had 0.43-times the risk (95% CI: 0.32–0.58) of having at least one missing value among nine required data elements compared to records from baseline RDQAs. Using a scale with one point awarded for each of 20 data elements with concordant values in paper records and KenyaEMR, we found that data concordance improved from baseline (11.9/20) to follow-up (13.6/20) RDQAs, with the mean concordance score increasing by 1.79 (95% CI: 0.25–3.33).

**Conclusions:**

This manuscript demonstrates that RDQAs can be implemented on a large scale and used to identify EMR data quality problems. RDQAs were associated with meaningful improvements in data quality and could be adapted for implementation in other settings.

## Introduction

Achievement of the ambitious UNAIDS 90-90-90 HIV care and treatment targets is dependent upon having strong health information systems with complete, accurate, and timely information that can be used to facilitate appropriate clinical care, monitor the HIV epidemic and progress towards targets, identify HIV hotspots, and help maximize the impact of the limited funding available for HIV prevention. UNAIDS 90-90-90 targets specify that three objectives will be achieved by 2020: 90% of all people living with HIV will know their HIV status, 90% of all people with diagnosed HIV infection will receive sustained antiretroviral therapy, and 90% of all people receiving antiretroviral therapy will have viral suppression. These targets are geared towards ending the AIDS epidemic by 2020 [1–3].

As tools that facilitates complete, accurate, and timely information, electronic medical record (EMR) systems have the potential to improve the quality of patient care, reduce the workload for healthcare workers, facilitate monitoring and evaluation of health programs, and provide timely data for decision-making [4–8]. In light of these potential benefits, significant financial investments have been made installing HIV care and treatment EMRs in low-resource settings [9], [10].

The benefits of EMRs are dependent upon the system having accurate, reliable, and timely data. Data quality, meaning data which is accurate, reliable, “fit for use” and relevant [11–14], is important for effective service delivery, decision making, and on-going monitoring of programs so that they maintain high quality of healthcare. Several studies have reported that data users’ lack of trust in the quality of AIDS, cancer, and health management information systems is due to unreliable or uncertain data [15–20]. EMR data quality has been found to be problematic in other settings [21–23], and because EMRs provide foundational health information which can be re-used to monitor and evaluate health services, poor data quality can cause facilities to understate their performance on programmatic indicators [24]. Problems may arise from non-intuitive user interfaces and other design issues, challenges around implementation such as inadequate training, or health system weakness beyond the EMR such as inadequate staffing or inconsistent electricity [21], [25], [26]. In light of these recognized challenges, it is essential for EMR systems to have routine activities in place that both monitor and improve data quality. While there is fairly robust evidence about the negative effects of poor data quality, there is less evidence about how to achieve strong data quality in routine health information systems, including EMRs.

Evaluations of EMRs in high-income settings have found that assessing data quality at facilities and providing feedback to system users can lead to meaningful improvements in data quality [27–29]. In low-resource settings, there is a need to develop evidence-based interventions to improve data quality that are inexpensive and sustainable. Wagenaar et al (2015) and Mphatswe et al (2012) describe positive results of interventions to strengthen routine data quality through training, audit and feedback in Mozambique and South Africa, respectively [30], [31]. However, neither study specifically focused on data quality improvement within EMRs. The World Health Organization recently published a data quality review toolkit for facility data quality assessment which offers common core indicators of data quality, guidelines for desk review, and methods for routine data quality assessments [32]. Such methodologic guidance must be paired with evidence that efforts invested in data quality assessment can not only illuminate problems, but also lead to improvement, including in health care settings where EMRs are central piece of the broader health information system.

Our focus was on testing the effectiveness of a data quality assessment and improvement process for EMRs. In Kenya, Routine Data Quality Assessment (RDQA) were developed to allow Ministry of Health (MOH) staff and HIV care and treatment implementing partners to efficiently assess the quality of EMR data and provide facility staff feedback during one-day site visits. RDQAs were envisioned to serve a dual purpose by obtaining information on the status of data quality at facilities, while also exposing data quality issues and spurring improvements in data quality. We tested the intervention of using repeated RDQAs in sites implementing KenyaEMR, an OpenMRS-based HIV care and treatment EMR system implemented across more than 300 facilities in Kenya since 2012.

RDQAs focused on two dimensions of data quality: data completeness and concordance. In our analysis, we assess the quality of data from facilities using KenyaEMR. Using data from baseline RDQAs, we explore how facility characteristics were associated with data quality prior to our intervention. We then assess if the feedback and action plan developed during the baseline RDQAs were associated with improvements in data quality by determining if there were changes in data quality between the baseline and follow-up RDQAs. Finally, we test the hypothesis that the feedback provided at baseline RDQAs led to improvements in data quality during follow-up RDQAs.

## Materials and methods

### KenyaEMR

KenyaEMR is an electronic medical records system that supports HIV care and treatment programs in Kenya. The system is customized from the OpenMRS platform (http://openmrs.org/). The Kenya MOH led KenyaEMR implementation within the national HIV/AIDS care and treatment program. The International Training and Education Center for Health (I-TECH), funded by PEPFAR through the U.S. Health Resources and Services Administration, supported the implementation of KenyaEMR at 341 health facilities across four regions of Kenya from 2012 to 2014. KenyaEMR was designed to support both retrospective and point of care data entry, with a majority of facilities equipped for point of care implementation. I-TECH, in close collaboration with the Kenya MOH and other PEPFAR partners, was tasked to provide training, technical support, and feature development for KenyaEMR.

The MOH-257 Comprehensive Care Clinic Patient Card (previously known as the Blue Card and now Green card) has historically been the paper record used to capture longitudinal HIV care and treatment information in Kenya. Facilities adopting KenyaEMR are initially required to record patient information in both the MOH-257 paper form and KenyaEMR. In addition, facilities are required to perform legacy data migration, where all historical data recorded on MOH-257 forms are retrospectively entered into KenyaEMR.

### Routine data quality assessments

To support progressive KenyaEMR data quality improvement, I-TECH worked with the MOH, HIV care and treatment implementing partners, and CDC to develop tools and standard operating procedures for routine data quality assessment (RDQA) and data-cleaning at facilities implementing KenyaEMR. RDQAs are led by county health authorities and implementing partners, with involvement of personnel in targeted facilities. When conducting an RDQA, assessment teams enumerate all clients who have ever received HIV care and treatment at the facility and randomly sample approximately 100 client records for review. Teams abstract values for 24 data elements related to HIV care and treatment services from both paper records (the MOH-257 Form) and KenyaEMR. The assessment focuses on two dimensions of data quality: data completeness and concordance [33]. Data completeness is assessed by determining the proportion of missing or invalid data for each data element, while concordance is assessed by comparing values between paper records and KenyaEMR.

A central component of the RDQA is an Excel-based tool into which data from the paper forms and KenyaEMR are abstracted. This tool automatically generates graphs showing the completeness and concordance for all 24 data elements, enabling teams to immediately assess the results, provide feedback to the facility staff, and develop action plans to improve data quality. It is envisioned that RDQAs will be repeated at facilities approximately every six months to consistently monitor data quality.

### Site selection

Facilities were considered eligible for an RDQA once they had completed legacy data migration. This is so because data concordance was assessed by comparing values between paper records and electronic records in KenyaEMR. As such, facilities qualified for an RDQA once they completed entering all the historic data into the EMR system. RDQAs included in this analysis were conducted over two time periods, with baseline RDQAs conducted from October to December 2014 and follow-up RDQAs conducted from June to September 2015. The county-level MOH staff and other PEPFAR implementing partners collaborated with I-TECH to determine which facilities received RDQAs.

Several factors influenced the likelihood that a facility received an RDQA, including convenience, recommendations of county officials, and prioritization by care and treatment implementing partners. The overarching criteria however remained the status of legacy data migration, namely whether facilities had completed 100% data migration from the paper records into the EMR system. In addition, the county-level MOH staff and other PEPFAR implementing partners prioritized some of the sites based on their workload, their enthusiasm towards the EMR as well as facilities requests to have an RDQA exercise. Notably, the RDQA activity was recommended for all the facilities implementing KenyaEMR as long as the legacy data was all keyed into the system. However, the exercise was conducted according to different timelines for different facilities depending on the factors mentioned above.

### Analysis

Data abstracted during the RDQAs using the Excel workbooks were imported into Stata version 13.1. Clients for whom either the paper or electronic record could not be located were excluded, as indicated by the abstracted record having missing values for all 24 data elements. The analysis of data accuracy included 20 of the 24 data elements reviewed during the RDQA: Patient ID, sex, date of birth, enrollment date, enrollment program, entry point, last visit date, next visit date, number of clinic visits, first CD4 count, last CD4 count, first WHO stage, last WHO stage, last co-trimoxazole date, ART start date, ART regimen, weight, transfer in date, transfer out date, and date of death. Four date values were excluded because their corresponding clinical values (first CD4, last CD4, first WHO stage, last WHO stage) were already included in the analysis and we felt that analyzing them would amount to double counting.

For each record reviewed during RDQAs, we generated a concordance score equal to the number of data elements with concordant values on the paper form and KenyaEMR. Data elements where both data sources reported missing values were considered to be concordant. A score of 20 indicates that all data elements for that record were concordant, while 0 indicates that no data elements were concordant. To identify factors associated with data concordance, we estimated the mean difference in concordance scores using generalized estimating equation (GEE) models with an identity link, normal distribution, exchangeable correlation matrix, and robust standard errors allowing for clustering by facility. The concordance score had an approximately normal distribution and was modeled as a continuous outcome, while facility-level factors hypothesized to be associated with the outcome were included as indicator variables in a multivariable model. A GEE model was selected to account for clustering by facility.

To assess data completeness, we identified nine mandatory data elements that should contain values for all individuals registered for HIV care and treatment. Though CD4 count, clinical staging, and co-trimoxazole provision are considered mandatory data elements by the Kenya MOH and should have been recorded for all clients, we excluded these data elements from our assessment of data completeness because we could not determine if the missing values were due to inaccurate documentation or failure by health providers to adhere to clinical guidelines. Since missing data across the nine data elements was uncommon, and the variable was not normally distributed, we created a binary variable for each record indicating if any of the nine data elements contained missing values. Using a GEE model with a log link, binomial distribution, exchangeable correlation matrix, and robust standard errors, we assessed if facility-level characteristics or the round of RDQA were associated with the risk of records having any missing data. Facility-level factors hypothesized to be associated with the outcome were included as indicator variables in a multivariable model, and a GEE model was used to account for clustering by facility.

## Results

**Fig 1:**
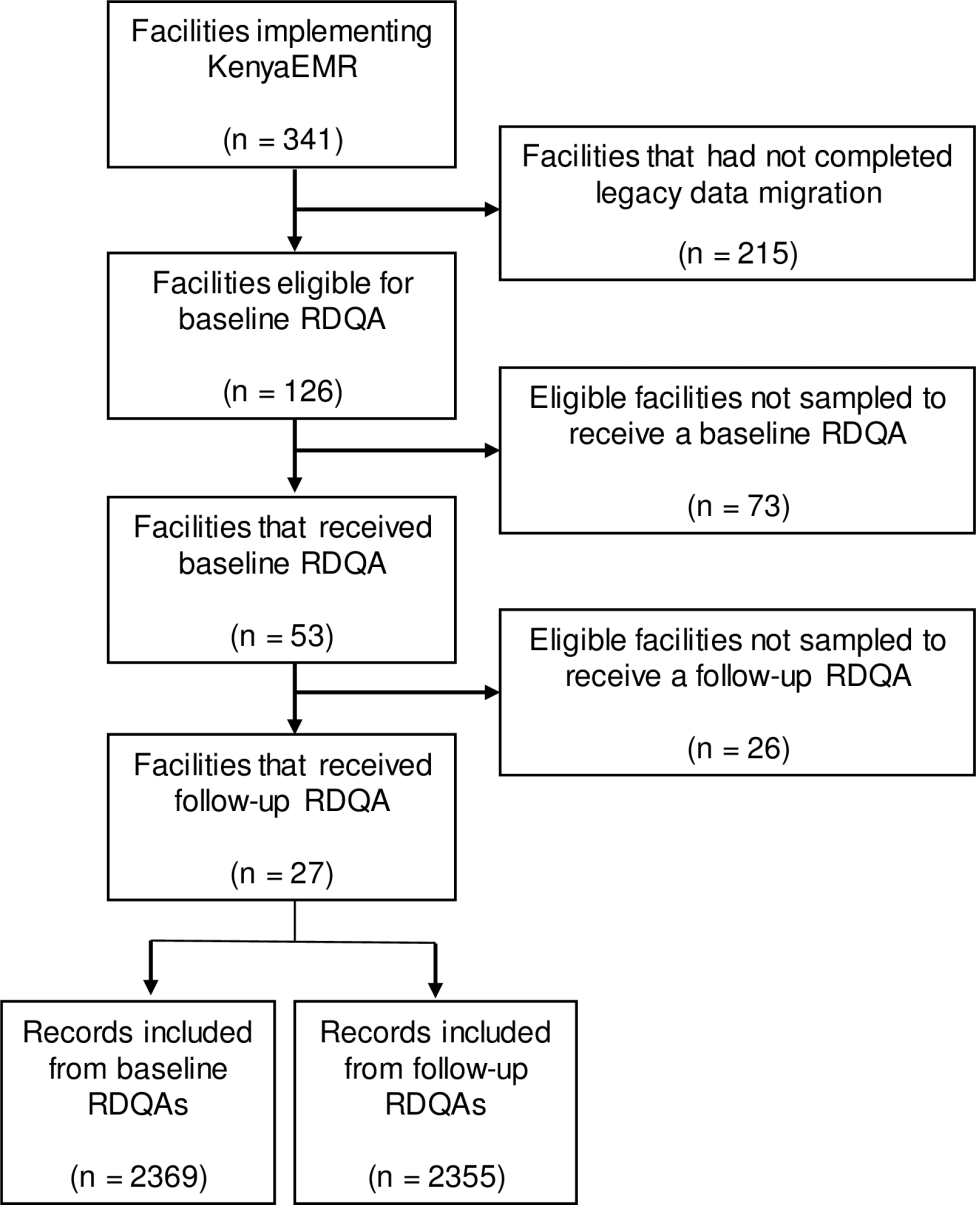
Flow diagram of facility selection for RDQA. RDQAs analysis included 27 facilities out 341 facilities implementing KenyaEMR.

Of the 341 facilities implementing KenyaEMR, approximately 126 facilities were eligible to receive a baseline RDQA since they had completed legacy migration of paper records into KenyaEMR. Fifty three of those 126 facilities had a baseline RDQA, with 27 of those 53 facilities receiving a follow-up RDQA. Of the 27 facilities assessed, 18 were health centres and nine were hospitals (Table 1). All facilities were equipped for point of care EMR implementation, though the level of point of care use varied across facilities. The Kenya MOH operated 24 of the facilities, while three facilities were operated by faith-based organizations. Facilities had been using KenyaEMR for between five and 23 months at the time of the baseline RDQAs, and the patient population who had ever enrolled in HIV care and treatment at facilities ranged from 145 to 16613 patients. Across the 27 facilities a total of 4792 client records were reviewed. 51 paper-based patient records (37 at baseline and 14 at follow-up) and 18 electronic-based patient records (6 at baseline and 12 at follow-up) could not be located and were excluded from further analysis. A single record could not be located among both the paper and electronic records. Thus, a total of 68 records were excluded, leaving 2369 records from baseline RDQAs and 2355 records from follow-up RDQAs in the analysis (Fig 1).

**Table 1:** Characteristics of the 27 facilities that participated in both baseline and follow-up RDQAs.

	Facilities n (%)	Patient records Baseline n (%)	Patient records Follow-up n (%)
**Facility type**			
Health centre	18 (67)	1482 (63)	1463 (62)
Hospital	9 (33)	887 (37)	892 (38)
**Facility ownership**			
Ministry of Health	24 (89)	2136 (90)	2140 (91)
Faith-based organization	3 (11)	233 (10)	215 (9)
**Months of EMR implementation**^1^			
5–8 months	7 (26)	531 (22)	544 (23)
12–15 months	12 (44)	1030 (43)	977 (41)
16–23 months	8 (30)	808 (34)	834 (35)
**Patients ever enrolled in HIV care**^1^			
Under 300	12 (44)	891 (38)	875 (37)
301–999	6 (22)	541 (23)	531 (23)
1000 and above	9 (33)	937 (40)	949 (40)

^1^ Months of EMR implementation and patients ever enrolled in HIV care were measured during the baseline RDQAs.

The frequency of missing data on paper records and in KenyaEMR was assessed for each data element during baseline and follow-up RDQA (S1 Table). Reviewing paper records from the baseline RDQAs, among the nine required data elements, enrollment program (1%), patient ID (1%), sex (2%), and number of clinic visits (2%) had the least missing data, while in KenyaEMR, sex (5%), date of birth (6%), patient ID (6%), and enrollment program (7%) had the least missing data. Entry point had the highest frequency of missing data in both paper records (12%) and in KenyaEMR (14%).

To identify facility characteristics associated with data completeness prior to our intervention, we used data collected during baseline RDQAs to assess if facility characteristics were associated with the proportion of records that contained one or more missing values across nine required data elements (Table 2). At least one missing value was observed in 735 (31%) paper forms and 747 (32%) KenyaEMR records. In a multivariable analysis, we did not find evidence that facility type, facility ownership, months of EMR implementation, or the number of patients ever enrolled in HIV care was associated with the risk that paper forms contained missing values. In contrast, the frequency of missing values in KenyaEMR was associated with months of EMR implementation and the number of patients ever enrolled in HIV care. Longer implementation of KenyaEMR at facilities was associated with a higher proportion of records containing missing data. Compared to facilities that had implemented KenyaEMR for 5–8 months, records from facilities that had used the EMR for 12–15 months were 1.31-times (95% CI: 0.73–2.34) more likely to have missing data while records from facilities that had used the EMR for 16–23 months were 1.99-times (95% CI: 1.03–3.83) more likely to have any missing data across nine required data elements. Facilities with moderate patient loads had the most complete data in KenyaEMR. Compared to facilities that had fewer than 300 patients ever enrolled in HIV care, records from facilities with 301–999 patients were 0.47-times (95% CI: 0.27–0.82) as likely to have missing data while records from facilities with over 999 patients were 0.73-times (95% CI: 0.42–1.26) as likely to have missing data across nine required data elements. Facility type and facility ownership were not associated with the frequency of missing data in KenyaEMR.

**Table 2:** The association between facility characteristics and the risk that records contained missing values during baseline RDQAs.

	Paper Forms					KenyaEMR				
	n/Total^1^	(%)	aRR^2^	(95% CI)	P-value	n/Total^1^	(%)	aRR^2^	(95% CI)	P-value
**Facility type**										
Health centre	429/1482	(29)	Ref	—	—	423/1482	(29)	Ref	—	—
Hospital	306/887	(34)	1.18	(0.76–1.84)	0.451	324/887	(37)	1.06	(0.76–1.49)	0.726
**Facility ownership**										
Ministry of Health	659/2136	(31)	Ref	—	—	664/2136	(31)	Ref	—	—
Faith-based organization	76/233	(33)	1.13	(0.70–1.83)	0.621	83/233	(36)	1.29	(0.77–2.17)	0.330
**Months of EMR implementation**										
5–8 months	159/531	(30)	Ref	—	—	134/531	(25)	Ref	—	—
12–15 months	286/1030	(28)	0.88	(0.53–1.47)	0.624	310/1030	(30)	1.31	(0.73–2.34)	0.361
16–23 months	290/808	(36)	1.21	(0.51–2.85)	0.661	303/808	(38)	1.99	(1.03–3.83)	0.040
**Patients ever enrolled in HIV care**										
Under 300	274/891	(31)	Ref	—	—	305/891	(34)	Ref	—	—
301–999	170/541	(31)	0.90	(0.31–2.60)	0.849	110/541	(20)	0.47	(0.27–0.82)	0.008
1000 and above	291/937	(31)	0.80	(0.38–1.65)	0.539	332/937	(35)	0.73	(0.42–1.26)	0.261
**All facilities**	735/2369	(31)	—	—	—	747/2369	(32)	—	—	—

^1^ The number of records with at least one missing value among the nine required data elements (n) divided by the total number of records from each category of facility. The nine required data elements were patient ID, sex, date of birth, enrollment date, enrollment program, entry point, last visit date, next visit date, and number of clinic visits.

^2^ Multivariable GEE models were used to determine if facility characteristics were associated with the proportion of records that contained a missing value for any of nine required data elements. GEE models used a log link, binomial distribution, exchangeable correlation matrix, and robust standard errors that allow for clustering by facility.

Data concordance between the paper records and KenyaEMR was determined for all data elements during baseline and follow-up RDQAs (S2 Table). Twenty of the 25 data elements had an increase in concordance from baseline to follow-up, while non-mandatory data elements tended to have the highest concordance due to the large proportion of records with concordant missing data. The mean concordance score across all facilities during baseline RDQAs was 11.9, indicating that on average 11.9 of the 20 data elements incorporated into the concordance score contained the same value in paper records and in KenyaEMR (Table 3). We did not find that facility type, facility ownership, the number of months that KenyaEMR had been implemented at the facility, or the number of patients ever enrolled in HIV care at the facility were associated with the baseline concordance score.

**Table 3:** The association between facility characteristics and the frequency of concordant data elements in paper records and KenyaEMR during baseline RDQAs.

	Concordance score^1^	β^2^	(95% CI)	P-value
**Facility type**				
Health centre	11.7	Ref	—	—
Other facility type	12.1	1.15	(-1.76–4.06)	0.439
**Facility ownership**				
Ministry of Health	11.9	Ref	—	—
Faith-based organization	11.6	-1.25	(-3.06–0.56)	0.176
**Months of EMR implementation**				
13–18 months	11.4	Ref	—	—
19–23 months	12.7	0.57	(-2.30–3.43)	0.699
24–31 months	11.1	-1.64	(-7.00–3.72)	0.549
**Patients ever enrolled in HIV care**				
Under 300	11.6	Ref	—	—
301–999	12.9	1.84	(-0.55–4.24)	0.131
1000 and over	11.5	0.27	(-3.80–4.34)	0.896
**All facilities**	11.9	—	—	—

^1^ 20 data elements were used to generate the concordance score, with one point awarded for each of the 20 elements that had matching values recorded on paper records and KenyaEMR (0 indicates no concordant elements, 20 indicates complete concordance). The 20 data elements were patient ID, sex, date of birth, enrollment date, enrollment program, entry point, last visit date, next visit date, number of clinic visits, first CD4 count, last CD4 count, first WHO stage, last WHO stage, last co-trimoxazole date, ART start date, ART regimen, weight, transfer in date, transfer out date, and date of death.

^2^ A multivariable GEE model was used to determine if facility characteristics were associated with a difference in the mean concordance score across 20 data elements. GEE models used an identity link, normal distribution, exchangeable correlation matrix, and robust standard errors that allow for clustering by facility.

To determine if RDQAs were associated with improvements in data completeness, we compared the frequency of missing data between the baseline and follow-up RDQAs. We did not find evidence of a change in the frequency of missing data on paper forms. However, we observed that in KenyaEMR the proportion of records with at least one missing value across nine required data elements decreased from 31% at baseline to 13% at the follow-up RDQAs. After adjusting for facility characteristics, we found that records from follow-up RDQAs had 0.43-times the risk (95% CI: 0.32–0.58) of having data elements with missing values compared to records from baseline RDQAs. In addition, we assessed if the frequency of data concordance differed between baseline and follow-up RDQAs. We determined that data concordance improved, with the mean concordance score increasing from 11.9 to 13.6. After adjusting for facility characteristics, we found a statistically significant improvement in the concordance score of 1.79 (95% CI: 0.25–3.33) between the baseline and follow-up RDQAs (Table 4).

**Table 4:** The relative risk of missing data and mean change in concordance score from the baseline to the follow-up RDQAs.

	Baseline N = 2411		Follow-up N = 2381		Unadjusted model			Adjusted model^1^		
**Relative risk of missing data**	**n**	**(%)^2^**	**n**	**(%)^2^**	**RR^3^**	**(95% CI)**	**P-value**	**RR^3^**	**(95% CI)**	**P-value**
Baseline to follow-up, paper forms	735	(30)	760	(32)	1.04	(0.79–1.38)	0.785	1.09	(0.84–1.41)	0.522
Baseline to follow-up, KenyaEMR	747	(31)	320	(13)	0.43	(0.32–0.58)	<0.001	0.43	(0.32–0.58)	<0.001
**Change in concordance score**	**Mean**	**(SD)^4^**	**Mean**	**(SD)^4^**	**β^5^**	**(95% CI)**	**P-value**	**β^5^**	**(95% CI)**	**P-value**
Baseline to follow-up	11.9	(4.0)	13.6	(4.2)	1.79	(0.25–3.33)	0.023	1.79	(0.25–3.33)	0.023

^1^ Adjusting for facility type, facility ownership, months of EMR implementation, and facility patient load.

^2^ The number (n) and percent of records with at least one missing value among nine required data elements.

^3^ GEE models were used to determine if RDQA round was associated with the proportion of records that had any missing values among nine required data elements. GEE models used a log link, binomial distribution, exchangeable correlation matrix, and robust standard errors that allow for clustering by facility.

^4^ 20 data elements were incorporated into the concordance score, with one point awarded for each of the 20 elements that had matching values recorded on paper records and KenyaEMR (0 indicates no concordant elements, 20 indicates complete concordance).

^5^ GEE models were used to determine if the mean concordance score changed from the baseline to follow-up RDQAs. GEE models used an identity link, normal distribution, exchangeable correlation matrix, and robust standard errors that allow for clustering by facility.

## Discussion

The results of this analysis support our hypothesis that repeated RDQAs lead to improvements in data quality in KenyaEMR. We believe that the efforts made to conceptualize the RDQAs and the systematic operationalization of the assessments ensured reliable and valid measurements of data quality. Assessing the concordance between paper records and KenyaEMR, we observed improvements in data concordance from the baseline to the follow-up RDQAs. In addition, we found that the completeness of data improved, with missing data found less frequently in follow-up compared to baseline RDQAs. Importantly, the reduction in records with missing data from 31% to 13% and the increase in data concordance score from 11.9 to 13.6 were statistically significant and represent meaningful improvements in data quality. The exploitation of the two different data sources containing the same information allowed us to identify conflicting data, exposing the status of data quality and providing the opportunity for resolution and data quality improvement. Our findings are consistent with other studies that found improved data quality following data quality assessment, audit and feedback [18], [29]–[31].

In the dawn of information technology systems such as EMRs, there is growing consensus that high-quality electronic data is an important prerequisite to achieving the full benefits of such systems. Data from EMRs can be used for feedback, a core activity for quality improvement. Leveraging EMRs could help ensure effective service delivery, decision making, care coordination and evaluation of the programs in order to maintain high quality of healthcare [34–38]. In Kenya, EMR data is used at the site level to provide patient care (e.g. identifying clients missing appointments and requiring follow-up) and it is also routinely pushed to databases at regional and national levels to monitor program progress and identify areas needing additional resources. However, it should be noted that improving data quality is necessary but not sufficient to achieve better outcomes or quality of care.

For HIV care and treatment programs in low-resource settings, the importance of EMRs is expected to increase as programs transition to differentiated care models in an attempt to improve the efficiency of HIV service delivery [39]. Though a number of quantitative and qualitative reports and program evaluations document best practices and lessons learned around the initial implementation of EMR systems in low-resource settings [40], [41], there is a paucity of research on ways to provide long-term, sustainable support to established EMR systems. This analysis found high rates of missing data and low data concordance at facilities that had implemented KenyaEMR for an average of 13 months at the time of the baseline RDQAs. This poor data quality highlights the need for ongoing assessment and support to improve EMR data quality. One of the approaches that we recommend towards ensuring that such efforts are sustained is through institutionalization of RDQAs. Counties or other local jurisdictions could consider having benchmarks that reflects the desired or acceptable level of data quality for each metric or data element, as recommended by WHO to ensure sound decisions based on sound data [32]. Facilities not meeting these standards would then receive an intervention to improve data quality, which could consist of additional training, on-site mentorship, and follow-up assessments to ensure that data quality improves.

This analysis found that facilities which had implemented KenyaEMR for longer periods of time had a higher frequency of missing data in their EMR system. We believe the most likely explanation for this finding is that at initial implementation of KenyaEMR, facilities receive extensive training and great emphasis is placed upon the importance of EMR use. However, over time, new staff may be deployed without receiving adequate training or user motivation to maintain the data quality of the EMR system thus data quality efforts may wane. These results highlight the need to provide ongoing support to EMR systems. Our findings that RDQAs were associated with improvements in data quality suggest that routine assessment of data quality that involves providing feedback to sites and developing action plans to address areas identified for improvement are useful steps that can be taken to improve data quality. Though the RDQA modality described in this paper requires a paper record gold standard to assess data concordance, the RDQA methodology could be adapted for settings without paper records by focusing on validity checks or data element agreement within the EMR [33], [42]. The fact that RDQAs were implemented by users of EMR data such as the MOH and care and treatment implementing partners, rather than an external body funded to conduct the data assessments, suggests that these efforts may be sustained.

This evaluation found that facilities with a medium-sized number of HIV care and treatment patients had the least missing data in KenyaEMR. We suspect that small sites might have lower frequencies of data completeness due to the infrequent practice that staff have in registering patients for care, while high volume sites may have lower frequencies of data completeness due to staff being overburdened. Given that high volume sites contribute the largest amount of data to national evaluations, it may be beneficial for these facilities to receive more frequent RDQAs or other interventions to improve data quality.

Finally, we would like to acknowledge that investing in the type of RDQA process highlighted in this evaluation is only one part of the investment required for EMR success. EMR implementation requires significant up-front investments in IT infrastructure, software design and development, training, clinic-level operating costs, and information technology support [43]. EMRs require a seamless power supply, yet facilities in many developed countries such as Kenya experience frequent power challenges that pose a major challenge to EMR use. Additionally, EMRs must meet the needs of end users in the clinical community. If an EMR is not sufficiently useful to users it can lead to apathy and poor system adoption.

## Limitations

There are a few limitations that should be considered when interpreting the results of this evaluation. First, the facilities selected for inclusion in this analysis were not randomly selected. Instead, we included all facilities that had received two RDQAs within our defined time-period. There could be bias arising in such selection since these facilities may happen to be the most zealous when it comes to EMR adoption and use and may have better data quality. The facilities included in the analysis may therefore not be representative of the entire population of 341 facilities using KenyaEMR; however, 21 of the 27 facilities experienced improvements in data concordance and all 27 facilities experienced a decrease in missing data, suggesting that improvements in data quality were widespread across facilities.

Additionally, this analysis did not include a control population of facilities that did not receive RDQAs, which would have enabled us to control for factors other than RDQAs that might influence data quality. We believe that the RDQAs were the probable cause of the observed improvements in data quality because it was the only widespread activity during the period of interest focused on improving the data quality in KenyaEMR. Another limitation of our analysis is that transcription errors may have occurred during the data abstraction process, which might result in an underestimation of the data quality in KenyaEMR, but we would not expect this effect to be differential between the baseline and follow-up RDQAs. Lastly, although we hypothesize that improved data quality has the potential to improve the quality of program implementation, it was not within the scope of this evaluation to assess if improved data quality led to improved patient outcomes. This would be an important association to evaluate in future studies.

Strengths of this analysis include the fact that RDQAs were performed by multiple stakeholders under routine implementation settings. A large number of records were evaluated from 27 different facilities with a diverse range of characteristics that were located across a broad geographic area of Kenya. RDQAs can be performed by staff with minimal training and can be conducted in a single day, making them relatively easy and inexpensive to implement. We believe that the standard operating procedure and set of tools for routine data quality assessment (RDQA) and data-cleaning could be easily modified for use in a range of different settings and if institutionalized and done routinely, have the potential to meaningfully improve data quality.

Improved outcomes can only be achieved when data are utilized to improve the quality of program implementation and patient care, and assessing if RDQAs led to improved outcomes was beyond the scope of this evaluation. Nonetheless, we believe that improved data quality will generally lead to better program implementation and improved patient outcomes. We recommend that future studies of data quality assessment and improvement efforts should examine the relationships between resulting data quality, provider perceptions of trust in the data, provider intended and actual use of data in patient and clinical management, and end results of quality of care and evidence-informed policy decisions, with a particular focus on EMRs.

## Conclusions

This evaluation demonstrated that RDQAs can be implemented on a large scale and were associated with meaningful improvements in EMR data quality. These findings suggest that RDQAs may be a useful method for improving the data quality of EMRs in low-resource settings. We identified frequent data quality problems when conducting the baseline round of RDQAs, so stakeholders that utilize data from other EMRs should evaluate the data quality in their system to determine if the data is fit for analysis and so that limitations of the data are understood. Organizations managing EMRs in other low-resources settings may want to implement activities such as RDQAs to improve the data used for decision-making. Future studies will need to evaluate the frequency at which RDQAs should be conducted at sites and assess if RDQAs, by improving data quality, are associated with better patient outcomes.

## Supporting information

S1 Table (PDF)

S2 Table (PDF)
