# Supplementary material for: The impact of routine data quality assessments on electronic medical record data quality in Kenya
Source: PLoS One. 2018 Apr 18;13(4):e0195362. doi: 10.1371/journal.pone.0195362 (PMC5905951; doi:10.1371/journal.pone.0195362)
Supplement: S1 Table — (PDF) [file pone.0195362.s001.pdf]

Table S1. Frequency of missing data for each data element

	Paper record		KenyaEMR	
	Baseline n (%) N=2369	Follow-up n (%) N=2355	Baseline n (%) N=2369	Follow-up n (%) N=2355
**Mandatory data elements**				
Patient ID*	30 (1)	45 (2)	134 (6)	1 (0)
Sex*	41 (2)	295 (13)	127 (5)	0 (0)
Date of birth*	206 (9)	359 (15)	133 (6)	0 (0)
Enrollment date*	230 (10)	405 (17)	238 (10)	0 (0)
Enrollment program*	24 (1)	156 (7)	174 (7)	0 (0)
Entry point*	285 (12)	468 (20)	329 (14)	165 (7)
Last visit date*	162 (7)	313 (13)	216 (9)	1 (0)
Next visit date*	159 (7)	456 (19)	324 (14)	184 (8)
Number of clinic visits*	51 (2)	124 (5)	249 (11)	1 (0)
1st CD4 count	720 (30)	809 (34)	1027 (43)	670 (28)
1st CD4 date	1098 (46)	836 (35)	1251 (53)	670 (28)
Last CD4 count	691 (29)	887 (38)	742 (31)	670 (28)
Last CD4 date	1172 (49)	889 (38)	1236 (52)	670 (28)
1st WHO stage	451 (19)	561 (24)	894 (38)	322 (14)
1st WHO date	968 (41)	578 (25)	1183 (50)	308 (13)
Last WHO stage	536 (23)	553 (23)	497 (21)	314 (13)
Last WHO date	1016 (43)	551 (23)	1000 (42)	302 (13)
Last CTX date	237 (10)	350 (15)	295 (12)	160 (7)
**Non-mandatory data elements**				
ART start date	752 (32)	876 (37)	789 (33)	732 (31)
ART regimen	623 (26)	806 (34)	789 (33)	738 (31)
Weight (at ART initiation)	975 (41)	1157 (49)	1434 (61)	1749 (74)
Transfer in date	2026 (86)	2092 (89)	2221 (94)	2032 (86)
Transfer out date	2255 (95)	2210 (94)	2247 (95)	2249 (95)
Death date	2333 (98)	2283 (97)	2241 (95)	2237 (95)

* The nine data elements that should have been completed for all individuals, regardless of clinical practice. The proportion of records with a missing value for any of the nine data elements was assessed.
