# Supplementary material for: The impact of routine data quality assessments on electronic medical record data quality in Kenya
Source: PLoS One. 2018 Apr 18;13(4):e0195362. doi: 10.1371/journal.pone.0195362 (PMC5905951; doi:10.1371/journal.pone.0195362)
Supplement: S2 Table — (PDF) [file pone.0195362.s002.pdf]

Table S2. Frequency of concordant data for each data element

	Discordant values		Concordant values			
			Data element value missing		Data element populated	
	Baseline n (%) N=2369	Follow-up n (%) N=2355	Baseline n (%) N=2369	Follow-up n (%) N=2355	Baseline n (%) N=2369	Follow-up n (%) N=2355
**Mandatory data element**						
Patient ID	1326 (56)	161 (7)	4 (0)	1 (0)	1039 (44)	2193 (93)
Sex	341 (14)	386 (16)	3 (0)	0 (0)	2025 (85)	1969 (84)
Date of birth	1237 (52)	1015 (43)	13 (1)	0 (0)	1119 (47)	1340 (57)
Enrollment date	1180 (50)	1013 (43)	45 (2)	0 (0)	1144 (48)	1342 (57)
Enrollment program	462 (20)	257 (11)	3 (0)	0 (0)	1904 (80)	2098 (89)
Entry point	1385 (58)	862 (37)	92 (4)	74 (3)	892 (38)	1419 (60)
Last visit date	1327 (56)	1211 (51)	30 (1)	0 (0)	1012 (43)	1144 (49)
Next visit date	1307 (55)	958 (41)	78 (3)	127 (5)	984 (42)	1270 (54)
Number of clinic visits	1731 (73)	1591 (68)	12 (1)	0 (0)	626 (26)	764 (32)
1st CD4 count	1126 (48)	933 (40)	562 (24)	503 (21)	681 (29)	919 (39)
1st CD4 date*	1082 (46)	1379 (59)	972 (41)	509 (22)	315 (13)	467 (20)
Last CD4 count	1224 (52)	973 (41)	430 (18)	530 (23)	715 (30)	852 (36)
Last CD4 date*	1101 (46)	1353 (57)	1002 (42)	531 (23)	266 (11)	471 (20)
1st WHO stage	1162 (49)	984 (42)	270 (11)	174 (7)	937 (40)	1197 (51)
1st WHO date*	961 (41)	1432 (61)	836 (35)	179 (8)	572 (24)	744 (32)
Last WHO stage	853 (36)	712 (30)	302 (13)	201 (9)	1214 (51)	1442 (61)
Last WHO date*	958 (40)	1162 (49)	859 (36)	199 (8)	552 (23)	994 (42)
Last CTX date	1320 (56)	1147 (49)	74 (3)	76 (3)	975 (41)	1132 (48)
**Non-mandatory data elements[1]**						
ART start date	927 (39)	731 (31)	546 (23)	608 (26)	896 (38)	1016 (43)
ART regimen	706 (30)	582 (25)	527 (22)	603 (26)	1136 (48)	1170 (50)
Weight (at ART start)	1068 (45)	986 (42)	795 (34)	1023 (43)	506 (21)	346 (15)
Transfer in date	321 (14)	207 (9)	1973 (83)	1975 (84)	75 (3)	173 (7)
Transfer out date	169 (7)	150 (6)	2176 (92)	2160 (92)	24 (1)	45 (2)
Death date	127 (5)	104 (4)	2227 (94)	2213 (94)	15 (1)	38 (2)

* Data elements not included in the concordance score.
